# Evaluation of SARS-CoV-2 IgG antibody response in PCR positive patients: Comparison of nine tests in relation to clinical data

**DOI:** 10.1371/journal.pone.0237548

**Published:** 2020-10-27

**Authors:** Paul Naaber, Kaidi Hunt, Jaana Pesukova, Liis Haljasmägi, Pauliina Rumm, Pärt Peterson, Jelena Hololejenko, Irina Eero, Piia Jõgi, Karolin Toompere, Epp Sepp

**Affiliations:** 1 SYNLAB Estonia, Tallinn, Estonia; 2 Department of Microbiology, Institute of Biomedicine and Translational Medicine, University of Tartu, Tartu, Estonia; 3 Kuressaare Hospital, Kuressaare, Estonia; 4 Molecular Pathology, Institute of Biomedicine and Translational Medicine, University of Tartu, Tartu, Estonia; 5 Department of Pediatrics, Institute of Clinical Medicine, University of Tartu, Tartu, Estonia; 6 Children's Clinic of Tartu University Hospital, Tartu, Estonia; 7 Department of Epidemiology and Biostatistics, Institute of Family Medicine and Public Health, University of Tartu, Tartu, Estonia; Qatar University, QATAR

## Abstract

SARS-CoV-2 antibody tests are available in various formats, detecting different viral target proteins and antibody subclasses. The specificity and sensitivity of SARS-CoV-2 antibody tests are known to vary and very few studies have addressed the performance of these tests in COVID-19 patient groups at different time points. We here compared the sensitivity and specificity of seven commercial (SNIBE, Epitope, Euroimmun, Roche, Abbott, DiaSorin, Biosensor) and two in-house LIPS assays (LIPS N and LIPS S-RBD) IgG/total Ab tests in serum samples from 97 COVID-19 patients and 100 controls, and correlated the results with the patients’ clinical data and the time-point the test was performed. We found a remarkable variation in the sensitivity of antibody tests with the following performance: LIPS N (91.8%), Epitope (85.6%), Abbott and in-house LIPS S-RBD (both 84.5%), Roche (83.5%), Euroimmun (82.5%), DiaSorin (81.4%), SNIBE (70.1%), and Biosensor (64.9%). The overall agreement between the tests was between 71–95%, whereas the specificity of all tests was within 98–100%. The correlation with patients’ clinical symptoms score ranged from strongest in LIPS N (ρ = 0.41; p<0.001) to nonsignificant in LIPS S-RBD. Furthermore, the time of testing since symptom onset had an impact on the sensitivity of some tests. Our study highlights the importance to consider clinical symptoms, time of testing, and using more than one viral antigen in SARS-CoV-2 antibody testing. Our results suggest that some antibody tests are more sensitive for the detection of antibodies in early stage and asymptomatic patients, which may explain the contradictory results of previous studies and should be taken into consideration in clinical practice and epidemiological studies.

## Introduction

Currently, more than 300 tests are available for SARS-CoV-2 antibody detection [1]. These tests are produced in several formats and they detect different types of antibodies including IgG, IgM or IgA subtypes or total immunoglobulin. In addition, the target proteins used to detect antibodies vary between the tests. Commercial tests are usually designed to detect antibodies against SARS-CoV-2 nucleocapsid (N), spike1 (S1), spike2 (S2), or receptor-binding domain of the spike (S-RBD) protein, or their combinations, though not all commercial providers specify the viral proteins used. Given the large variability in antibody tests, discrepancies between test results are expected. Concordantly, at the moment no agreement exists upon which viral protein should be used as a gold standard in serodiagnosis of COVID-19 patients.

Although the producers have usually reported high sensitivity and specificity for their tests, variable clinical sensitivity has been reported by independent studies [2–5]. However, minimal data is available on their sensitivity and specificity for their differences in target proteins.

The majority of clinical studies and validations of commercial tests have been performed in patient groups with severe disease and thus reported sensitivity data may not be the same for COVID-19 patients with mild symptoms. Only a few studies have investigated the antibody responses in pauci-symptomatic or asymptomatic persons [6, 7]. Several studies have shown stronger antibody response in patients with severe disease as compared with mildly symptomatic ones. Also higher rate of absence of seroconversion in asymptomatic patients has been described. However, other studies have failed to find any correlation between clinical course and immune response [3, 6]. Since the majority of COVID-19 cases are asymptomatic, the performance of the tests in this group is important to evaluate the reliability of antibody tests in seroepidemiological studies and clinical diagnostics [8].

It is known that the sensitivity of the antibody test depends on sampling time. Different studies have reported variable time of appearance of antiviral IgG antibodies but in most publications the median seroconversion time has been between 6 and 14 days from symptoms onset. Although several studies have shown high IgG at least for seven weeks, rapid decline of IgG in convalescence phase has been reported in asymptomatic COVID-19 patients [2, 3, 6]. It seems, that the optimal time for IgG detection (with the highest sensitivity rate) may also depend on clinical course of COVID-19 and is not clearly defined yet.

There is variation in antibody tests design (different target viral proteins has been used) on the one hand, and the conflicting results of clinical studies (tests sensitivities, antibody response dependence on clinical cause, optimal testing time) on the other hand. Thus, there is lacking information on how different SARS-CoV-2 antibody tests perform in subgroups of COVID-19 patients and at variable time points.

Our study aimed to compare the performance characteristics of seven commercial and two in-house IgG/total Ab tests, which analyze the reactivity to several target proteins, and to correlate the results with the patients’ clinical data (with different symptoms score and age), and time from disease onset.

## Material and methods

### Ethics statement

The Study has been approved by Research Ethics Committee of the University of Tartu on April 23, 2020 (nr 311/T-1). Patients signed informed consent before recrutement into the study.

### Patients’ recruitment and sample collection

Serum samples from 97 persons with COVID-19 were collected between April 28 and May 07 2020 from Kuressaare hospital located on the island of Saaremaa in Estonia. Persons who had been diagnosed COVID-19 by positive SARS-CoV-2 RT-PCR regardless of clinical symptoms were invited to participate. These persons included hospitalized and ambulatory patients as well as healthy contacts of confirmed COVID-19 cases selected randomly. The time from symptoms onset or positive PCR to serum collection had to be at least one week. Included persons signed informed consent agreeing with sampling and usage of clinical data. Medical personnel in Kuressaare hospital performed blood sampling (10ml from each patient), recorded time of clinical onset and patients’ symptoms related to COVID-19 using modified WHO questionnaire. Samples and patients’ data were sent to SYNLAB Estonia central laboratory in coded manner.

Patients were scored based on a number of different symptoms present during COVID-19 episode. On that basis we classified patients as asymptomatic (no symptoms before or after positive SARS-CoV-2 RT-PCR test), patients with 1–6 symptoms and patients with ≥7 different symptoms.

Serum was separated and aliquoted before storage. All aliquots were stored at– 30° C and analyzed within one month applying one freezing/thawing cycle before testing.

For testing the specificity of applied tests, 100 anonymous serum samples collected before COVID-19 pandemic and stored in SYNLAB Estonia were used. These samples were taken from healthy persons for various health control laboratory tests and have not been screened for virus related antibodies.

### Tests

Five laboratory tests for IgG (SNIBE, Euroimmun, Abbott, Epitope, DiaSorin), one laboratory total Ab (Roche) test, one rapid IgG test (SD Biosensor) and 2 in-house IgG tests (LIPS N and LIPS S-RDB) were compared. Different protein markers have been used in these tests (S1, S2, S-RBD, N or their combinations). Detailed information about the tests is summarized in S1 Table. Commercial tests were performed and interpreted according to manufacturer instruction, in-house LIPS as described previously [9].

### Statistics

The following analysis was made: correlation by Spearman's test, comparison of qualitative data (positivity rate) by Fisher exact test, groups’ comparison by Kruskal-Wallis test, pairwise comparison by Conover-Iman test with Holm-Bonferron correction using Past 4.03 and Stata 14.2 software.

## Results

### COVID-19 patients

According to medical history and patients’ anamnesis, 20 patients (20.6%) had no symptoms before or after the positive PCR test. In 43 patients (44%), one to six different symptoms has been recorded, and 34 patients (35%) had seven or more symptoms related to COVID-19 episode. Detailed clinical data is shown in Table 1.

**Table 1:** COVID-19 patients (n = 97) clinical data.

Patients clinical data		Values
**Patients’ data**		
	Age in years (median; min-max)	59 (21–100)
	Male/Female ratio (%)	32/68
	TTT^a^ in days (median; min-max)	28 (7–57)
	Symptoms score^b^ (median; min-max)	4 (0–14)
	Hospitalization (%)	19.6
**Symptoms (%)**		
	Fever	43.3
	Chills	39.2
	Fatigue	59.8
	Muscle ache	37.1
	Sore throat	27.8
	Cough	45.4
	Rhinitis	37.1
	Difficult breathing	20.6
	Shortness of breath	16.5
	Chest distress	18.6
	Headache	45.4
	Nausea and vomiting	28.9
	Abdominal pain	11.3
	Diarrhea	26.8
	Other symptoms	5.2
**Asymptomatic (%)**		20.6

^a^TTT (Time to test) was calculated as days to serum sampling from symptoms onset in symptomatic cases or days from the first positive PCR in asymptomatic cases.

^b^Symptoms score = number of different symptoms related to COVID-19 episode recorded per person.

### Comparison of specificity by different tests

We found no false-positive results in testing 100 pre-COVID-19 sera by Roche, Abbott and Biosensor tests. Other tests gave one to two false positive results. Additionally, Euroimmun test gave 2% (2/100) and Epitope 4% (4/100) borderline results with pre-COVID-19 sera. The specificity and sensitivity of applied tests is shown in Table 2. In total, 15% (15/100) of control samples gave false positive results by any test. In most cases, only one test was positive per sample but 2% (2/100) of samples showed positive/borderline results by two (Epitope and Euroimmun) or three (SNIBE, Epitope, LIPS S-RBD) tests. The distribution of quantitative values of controls is presented in S1 Fig.

**Table 2:** Sensitivity and specificity of tests according to present study.

Test, manufacturer^a^	Antibody class and protein	Sensitivity^b^	Specificity^c^
MAGLUMI 2019-nCoV IgG, **SNIBE** (Shenzhen New Industries Biomedical Engineering Co)	IgG, not specified	70.1%	98%
SARS-CoV-2 ELISA IgG, **EUROIMMUN** AG	IgG, S1	82.5%	98%
SARS-CoV-2 IgG, **Abbott** Laboratories	IgG, N	84.5%	100%
Elecsys® Anti-SARS-CoV-2, **Roche** Diagnostics GmbH	Total Ab, N	83.5%	100%
EDI™ Novel Coronavirus COVID-19 IgG ELISA, **Epitope** Diagnostics Inc	IgG, N and S	85.6%	98%
LIAISON® SARS-CoV-2 S1/S2 IgG, **DiaSorin** S.p.A.	IgG, S1 and S2	81.4%	99%
STANDARDTM Q COVID-19 IgM/IgG Duo Test, SD **Biosensor** Inc	IgG, N	64.9%	100%
**LIPS S-RBD** IgG, in-house	IgG, S-RBD	84.5%^d^	98%^d^
**LIPS N** IgG, in-house	IgG, N	91.8%^d^	98%^d^

^a^Short names used in the text are indicated in bold.

^b^Based on testing of 97 serum samples from COVID-19 patients’ in present study.

^c^Based on testing of 100 pre COVID-19 sera in present study.

^d^In LIPS tests statistical (not clinically validated) cut-offs were applied.

### Comparison of sensitivity by different tests in COVID-19 patient

Out of 97 COVID-19 patients’ samples, 53 (55%) were positive and two (2%) negative by all nine tests used. Results varied by tests in the case of 42 (43%) samples. Sensitivity by different tests is shown in Table 2. The highest positivity rate was found in in-house LIPS N test (91.8% cases) and lowest in Biosensor rapid test (64.9%). The distribution of quantitative test values of COVID-19 cases in comparison with control samples is presented in S1 Fig.

The agreement between the tests (using qualitative interpretations) ranged between 95% (Euroimmun and DiaSorin) and 71% (LIPS N and Biosensor). The correlation between quantitative results was significant in all test combinations (p<0.001) except between in-house LIPS N and LIPS S-RBD that gave non-significant results. The strongest correlation was found between Euroimmun and DiaSorin tests (ρ = 0.95). The agreement between qualitative results and the correlation between quantitative values is shown in Table 3 and S2 Fig.

**Table 3:** Agreement between qualitative results (positive or negative) and correlation between quantitative values of different tests in COVID-19 patients’ samples (n = 97).

Tests	Agreement between qualitative results, %							
	Correlation between quantitative values, ρ (p<0.001 in all significant cases)							
	Epitope	Euroimmun	Roche	Abbott	DiaSorin	LIPS S-RBD	LIPS N	Biosensor
**SNIBE**	85	77	85	86	80	84	76	85
	0.91	0.64	0.68	0.84	0.70	0.6	0.56	NA^a^
**Epitope**		82	90	93	84	91	86	77
		0.56	0.59	0.80	0.65	0.53	0.46	NA^a^
**Euroimmun**			87	81	95	88	85	74
			0.66	0.65	0.95	0.73	0.50	NA^a^
**Roche**				89	88	91	88	79
				0.78	0.71	0.67	0.51	NA^a^
**Abbott**					82	90	87	78
					0.71	0.59	0.51	NA^a^
**DiaSorin**						87	84	77
						0.78	0.53	NA^a^
**LIPS S-RBD**							80	76
							NS^b^	NA^a^
**LIPS N**								71
								NA^a^

^a^ NA–not applicable. Only qualitative interpretation (absence or presence of test line).

^b^NS–no significant correlation (p>0.05).

### Relations between antibody detection and COVID-19 patients’ symptoms

Comparing patients’ symptom scores and the tests’ quantitative values, we found a significant positive correlation in all cases except with LIPS S-RBD. The strongest correlation was found in LIPS N (ρ = 0.41; p<0.001), followed by Roche (ρ = 0.39; p<0.001), Abbott and SNIBE (both ρ = 0.32; p = 0.001), DiaSorin (ρ = 0.31; p = 0.002), Epitope (ρ = 0.30; p = 0.003) and Euroimmun (ρ = 0.29; p = 0.004). The differences in quantitative test values and qualitative results among patient groups according to the number of symptoms are presented in Table 4.

**Table 4:** Antibody detection by tests in COVID-19 patients (n = 97) with different symptoms scores.

Tests	Quantitative test value median (25%; 75% percentiles)		
	% of positive tests in subgroup		
	Asymptomatic	Symptoms score 1–6	Symptoms score 7–14
	n = 20	n = 43	n = 34
**SNIBE**	0.79 (0.16; 12.6)^1^	2.39 (0.97; 12.11)	9.05 (2.00; 21.7)^1^
	40^a^^,^^b^	74^a^	82^b^
**Epitope**	0.47 (0.26; 0.90)^2^	0.61 (0.35; 0.83)	0.81 (0.45; 1.05)^2^
	80	86	88
**Euroimmun**	0.32 (0.65; 5.21)	4.60 (1.92; 7.25)	6.19 (2.45; 7.29)
	65	86	88
**Roche**	2.94 (0.45; 12.27)^3^^,^^4^	12.55 (3.02; 39.2)^3^	34.17 (6.34; 43.26)^4^
	60^c^^,^^d^	88^c^	91^d^
**Abbott**	3.02 (1.99; 5.21)^5^	5.61 (2.01; 7.65)^6^	6.82 (4.75; 7.98)^5^^,^^6^
	80	81	91
**DiaSorin**	37.00 (5.99; 67.80)^7^^,^^8^	70.40 (26.5; 134)^8^	86.1 (35.9; 154)^8^
	65	83	88
**LIPS S-RBD**	4.78 (2.14; 22.03)	14.19 (4.63; 58.10)	14.36 (6.03; 30.86)
	75	83	91
**LIPS N**	5.20 (2.83; 10.49)^9^^,^^10^	11.33 (6.24; 24.74)^9^^,^^11^	19.49 (9.12; 80.20)^10^^,^^11^
	80	95	94
**Biosensor**	NA^g^	NA^g^	NA^g^
	40^e^^,^^f^	65^e^	79^f^

Statistical difference between quantitative data

^1^p = 0.01

^2^p = 0.026

^3^p = 0.006

^4^p<0.001

^5^p = 0.004

^6^p = 0.04

^7^p = 0.017

^8^p = 0.008

^9^p = 0.018

^10^p<0.001

^11^p = 0.029, and between percentage of positive results

^a^p = 0.01

^b^p = 0.002

^c^p = 0.02

^d^p = 0.01

^e^p = 0.002

^f^p = 0.007

^g^NA–not applicable. Only qualitative interpretation (absence or presence of test line).

### Relations between antibody detection and time to test (TTT)

We found a significant correlation between TTT and the quantitative results of the test in case of Roche (ρ = 0.38; p<0.001), Euroimmun (ρ = 0.21; p = 0.04) and LIPS N (ρ = 0.28; p = 0.009) test. We also found significant differences between TTT groups in quantitative test data as well as in positivity rate in case of some but not all tests (Table 5).

**Table 5:** Antibody detection by tests in COVID-19 patients (n = 97) in different time to test groups.

Tests	Quantitative test value median (25%;75% percentiles)		
	% of positive tests in subgroup		
	7–14 days	15–30 days	31–57 days
	n = 20	n = 35	n = 42
**SNIBE**	2.02 (0.23; 17.51)	4.21 (0.8; 17.94)	5.18 (1.19; 14.02)
	55	69	79
**Epitope**	0.65 (0.3; 1.03)	0.73 (0.37; 0.96)	0.62 (0.36; 0.87)
	80	89	86
**Euroimmun**	2.43 (0.68; 4.92)^1^^,^^2^	5.18 (2.32; 7.88)^1^	4.81 (2.40; 6.83)^2^
	55^a^^,^^b^	89^a^	90^b^
**Roche**	3.18 (1.15; 8.24)^3^^,^^4^	15.05 (6.02; 51.45)^3^	31.90 (5.39; 43.16)^4^
	75	83	88
**Abbott**	4.27 (2.23; 6.77)	6.15 (2.67; 7.76)	5.88 (3.11; 7.65)
	85	86	83
**DiaSorin**	27 (6.46; 73.55)^5^^,^^6^	98.8 (28.7; 198)^5^	70.15 (30.2; 134)^6^
	55^c^^,^^d^	86^c^	90^d^
**LIPS S-RBD**	3.05 (1.85; 6.08)^7^^,^^8^	22.69 (9.31; 59.60)^7^	13.48 (4.68; 34.15)^8^
	70	91	86
**LIPS N**	10.49 (4.14; 35.73)	8.48 (3.38; 13.11)^9^	16.13 (9.12; 63.90)^9^
	90	89	95
**Biosensor**	NA^e^	NA^e^	NA^e^
	60	69	64

Statistical difference between quantitative data

^1^p = 0.006

^2^p = 0.01

^3^p = 0.003

^4^p<0.001

^5^p = 0.003

^6^p = 0.01

^7^p<0.001

^8^p<0.001

^9^p = 0.005, and between percentage of positive results

^a^p = 0.008

^b^p = 0.003

^c^p = 0.02

^d^p = 0.003

^e^NA–not applicable. Only qualitative interpretation (absence or presence of test line).

### Relations between antibody detection and patients’ age and sex

No correlations between test results and patients’ age or sex were found.

## Discussion

This is the first study evaluating the performance characteristics of several commercial and in-house SARS-CoV-2 antibody tests in different patient groups and different time to test points.

We found that different tests gave diverse antibody results if applied to heterogeneous COVID-19 patient group. In less than 60% of COVID-19 cases, all tests gave identical positive or negative result. Considering that all COVID-19 patients (confirmed by positive SARS-CoV-2 PCR test) should develop IgG antibodies, the sensitivity of tests varied from 65 to 92%, which is much less than reported by the manufacturers [1]. In agreement, similar low sensitivity rates have been reported in a recent meta-analysis on antibody tests [2]. A combination of two tests with different protein markers may increase sensitivity. For example according to our results re-testing Abbott IgG (N protein) negatives with index value 0.3–1.39 by DiaSorin IgG (S1 and S2 proteins), as practiced in SYNLAB Estonia, increases sensitivity by ca 7%. Although this combination did not affect the specificity in our control group, comparisons in larger study groups should be done. However, even if several different tests are combined, some confirmed COVID-19 cases remained negative for antibodies.

Correlation and agreements between the tests studied here varied highly in our COVID-19 group. The best correlation was found between Epitope and DiaSorin, which could be explained by detecting the antibodies to the same viral antigen (spike protein). One of the weakest agreements in positive/negative results and absence of correlation in quantitative test values was found when comparing IgG antibodies to nucleocapsid and to RBD of spike protein detected by LIPS tests. It is thus plausible that the patients develop diverse IgG antibody reactivities to SARS-CoV-2 proteins and their epitopes.

While analyzing the results from different tests in patient groups with different symptoms scores we found that patients with more different symptoms usually had a higher positivity rate and higher levels of antibodies. This is in accordance with some previous studies [3]. However, this relationship was not uniform in all tests. For example, LIPS detected significant differences in anti-N IgG levels between asymptomatic, pauci-symptomatic and poly-symptomatic COVID-19 patients. At the same time anti-RBD IgG variation within the patients groups was higher and no significant differences between groups were found. According to our data production of IgG antibodies against some virus proteins are more symptom-dependent than others.

Thus, according to our study, patients with more symptoms develop a higher immune response against virus proteins than asymptomatic ones. This makes the diagnosing of previous COVID-19 patients by antibody test more difficult in asymptomatic and pauci-symptomatic patients since the sensitivity of several tests is much lower in this subgroup than in highly symptomatic group. Since the majority of COVID-19 patients are asymptomatic or have only a few mild symptoms the sensitivity of antibody tests to detect disease in the general population could be lower [8]. This may affect the reliability of antibody-based epidemiological studies.

However, for some tests (such as Abbott), the absence of clinical severity seems not to affect positivity rate so much than for others (such as Biosensor rapid test or SNIBE) where the positivity rate in asymptomatic COVID-19 cases was about two times lower than in polysymptomatic ones. More studies are needed to confirm the finding that some antibody tests (that use specific antigens) are more suitable to diagnose asymptomatic COVID-19 cases than others.

We also found test-dependent differences while comparing antibody detection among different TTT groups. For example, anti-N IgG was detected by Abbott in high level already at 7–14 days TTT group and no differences were found with later TTT groups. In some other test (such as Euroimmun) lower detection rate and antibody levels were associated with shorter TTT. The reason could be in an analytical sensitivity of the test and also in the usage of different viral proteins–antibodies to some virus proteins may appear earlier and at increased levels than others.

Our study has several limitations. Firstly, although the onset of disease could be dated relatively precisely in symptomatic cases, in asymptomatic cases the disease detection depends on random PCR screening of risk groups. Thus, the first positive SARS-CoV-2 PCR is not equivalent to disease onset and one should be careful drawing conclusions based of TTT in such heterogeneous group of patients that includes symptomatic and asymptomatic patients. Secondly, we only analyzed IgG and total antibody values. The main reasons were the absence of IgM tests from several manufacturers (Abbott, DiaSorin) in the time of testing and questionable reliability of available ones. SNIBE and Epitope IgM tests gave significantly lower positive results than IgG tests and added no additional positive cases (all IgM positive cases were also IgG positive). Euroimmun IgA had a high positivity rate (ca 90% in COVID-19 patients) but also a high proportion of nonspecific reactions (21% of positive and borderline cases) in pre COVID-19 control group. Thus, until reliable commercial IgM and IgA tests are available, we can’t evaluate the role of these antibodies in infection and applicability of these tests in clinical practice or surveillance studies.

In conclusion, our study gave new theoretical insight to COVID-19 diagnostic testing and has practical implications. We confirmed that SARS-CoV-2 antibody response depends on clinical symptoms and time of testing, but we also found that this relation is dependent on test type and viral antigens used in the tests. This means that not all antibody tests work uniformly well in symptomatic and asymptomatic cases and in different time periods from disease onset. This explains some contradictory results of previous studies and should be taken into consideration in clinical practice and epidemiological studies.

## Supporting information

S1 Table Detailed information about applied SARS-CoV-2 antibody tests. (PDF)

S1 Fig Distribution of quantitative results of SARS-CoV-2 antibody tests: COVID-19 patients (n = 97) *vs* controls (n = 100). (PDF)

S2 Fig Correlations between SARS-CoV-2 antibody tests of COVID-19 patients (n = 97): Quantitative results plots. (PDF)
