# Supplementary material for: Evaluation of SARS-CoV-2 IgG antibody response in PCR positive patients: Comparison of nine tests in relation to clinical data
Source: PLoS One. 2020 Oct 27;15(10):e0237548. doi: 10.1371/journal.pone.0237548 (PMC7591045; doi:10.1371/journal.pone.0237548)
Supplement: S1 Table — (PDF) [file pone.0237548.s001.pdf]

S1 Table. Detailed information about applied SARS-CoV-2 antibody tests

**Test, manufacturer**	**Analyzer**	**Method**	**Protein marker reported by producer**	**Interpretation criteria**
MAGLUMI 2019-nCoV IgG, **SNIBE*** (Shenzhen New Industries Biomedical Engineering Co)	MAGLUMI 1000, SNIBE	Chemiluminescence immunoassay (CLIA)	Recombinant antigen, not specified	<1 neg; >1 pos
SARS-CoV-2 ELISA IgG, **EUROIMMUN*** AG	AGILITY®, Dynex Technologies	Enzyme immunoassay (ELISA)	Spike1 (S1)	<0.8 neg; ≥0.8 - <1,1 borderline; ≥1,1 pos
SARS-CoV-2 IgG, **Abbott*** Laboratories	ARCHITECT i2000SR, Abbott Laboratories	Chemiluminescent microparticle immunoassay (CMIA)	Nucleocapsid (N)	<1.4 neg; ≥1.4 pos
Elecsys® Anti-SARS-CoV-2, **Roche*** Diagnostics GmbH	Cobas® e411, Roche Diagnostics GmbH	Electrochemiluminescence immunoassay (ECLIA)	Nucleocapsid (N)	<1 neg; ≥1 pos
EDI™ Novel Coronavirus COVID-19 IgG ELISA, **Epitope*** Diagnostics Inc	AGILITY®, Dynex Technologies	Enzyme immunoassay (ELISA)	Nucleocapsid (N) and Spike (S)	neg 0.9 x (neg control + 0.10)*;* pos 1.1 x (neg control + 0.10)
LIAISON® SARS-CoV-2 S1/S2 IgG, **DiaSorin*** S.p.A.	LIAISON® XL Analyzer, DiaSorin S.p.A.	Chemiluminescence immunoassay (CLIA)	Spike1 (S1) and Spike2 (S2)	<12 neg; ≥12 - <15 borderline; ≥15 pos
STANDARDTM Q COVID-19 IgM/IgG Duo Test, SD **Biosensor*** Inc	Manual evaluation	Rapid chromatographic immunoassay	Nucleocapsid (N)	Positive: any line in test window
**LIPS S-RBD*** IgG, in-house	Victor™ X Plate reader, PerkinElmer Inc	Luciferase Immunoprecipitation Systems (LIPS) assay	Receptor binding domain of the spike (S-RBD)	Neg controls average + 3SD (2.1056 in this study)**
**LIPS N*** IgG, in-house	Victor™ X Plate Reader, PerkinElmer Inc	Luciferase Immunoprecipitation Systems (LIPS) assay	Nucleocapsid (N)	Neg controls average + 3SD (1.6723 in this study)**

* Short names used in text are indicated in bold; **Statistical cut-off, not clinically validated
