# Supplementary material for: Evaluation of SARS-CoV-2 IgG antibody response in PCR positive patients: Comparison of nine tests in relation to clinical data
Source: PLoS One. 2020 Oct 27;15(10):e0237548. doi: 10.1371/journal.pone.0237548 (PMC7591045; doi:10.1371/journal.pone.0237548)
Supplement: S1 Fig — (PDF) [file pone.0237548.s002.pdf]

S1 Fig. Distribution of quantitative results of SARS-CoV-2 antibody tests: COVID-19 patients (n=97) vs controls (n=100).

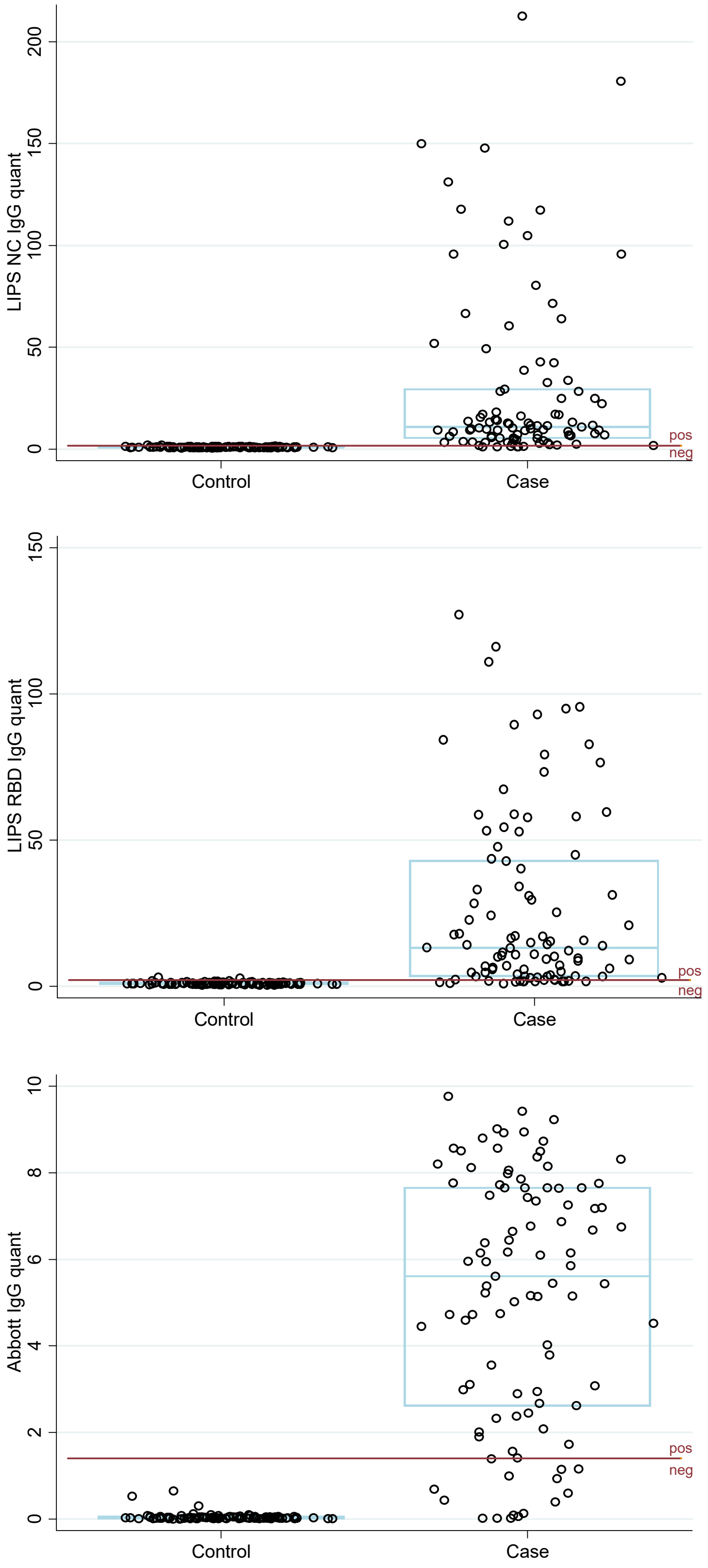

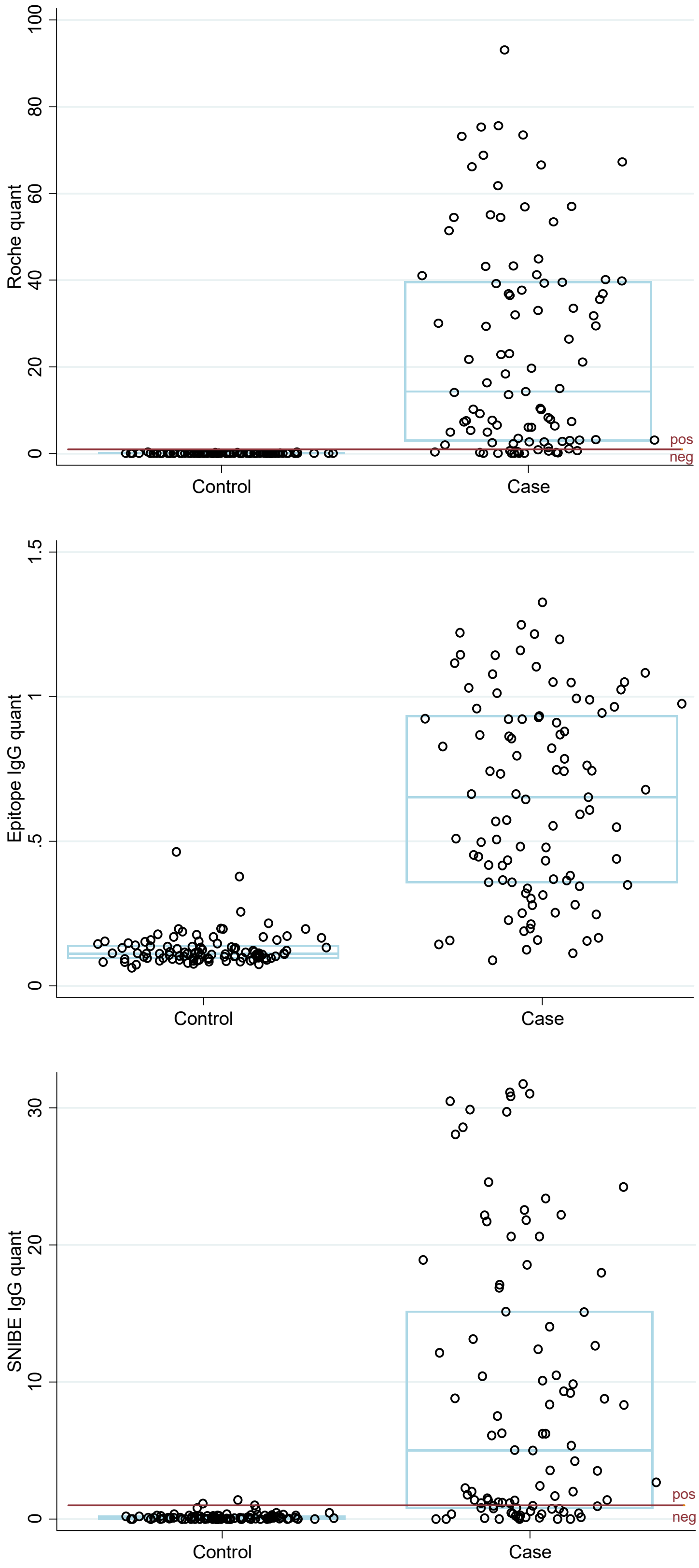
Roche quant
100
80
60
40
20
0
pos
neg
Control
Case
Epitope IgG quant
1.5
1
.5
0
Control
Case
SNIBE IgG quant
30
20
10
0
pos
neg
Control
Case

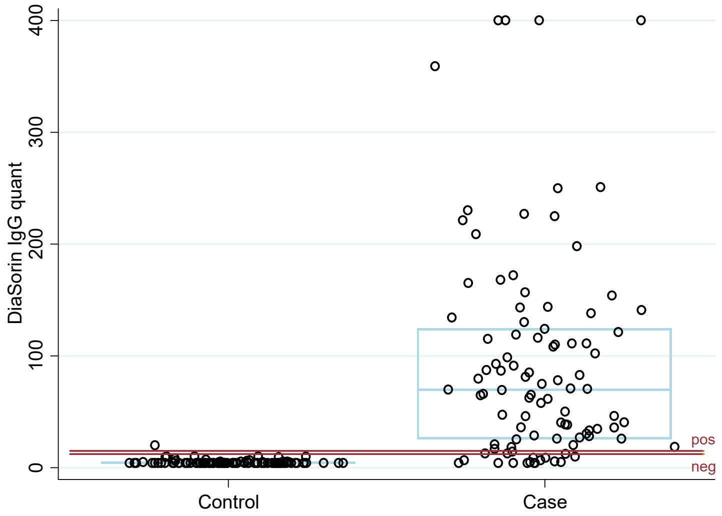

DiaSorin IgG quant
0
100
200
300
400
pos
neg
Control
Case

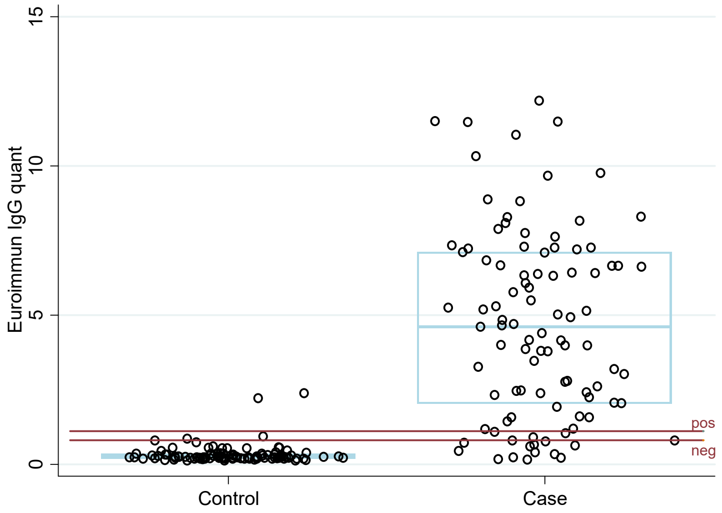

Euroimmun IgG quant
0
5
10
15
pos
neg
Control
Case
