# Supplementary material for: Evaluation of SARS-CoV-2 IgG antibody response in PCR positive patients: Comparison of nine tests in relation to clinical data
Source: PLoS One. 2020 Oct 27;15(10):e0237548. doi: 10.1371/journal.pone.0237548 (PMC7591045; doi:10.1371/journal.pone.0237548)
Supplement: S2 Fig — (PDF) [file pone.0237548.s003.pdf]

S2 Fig. Correlations between SARS-CoV-2 antibody tests of COVID-19 patients (n=97): quantitative results plots.

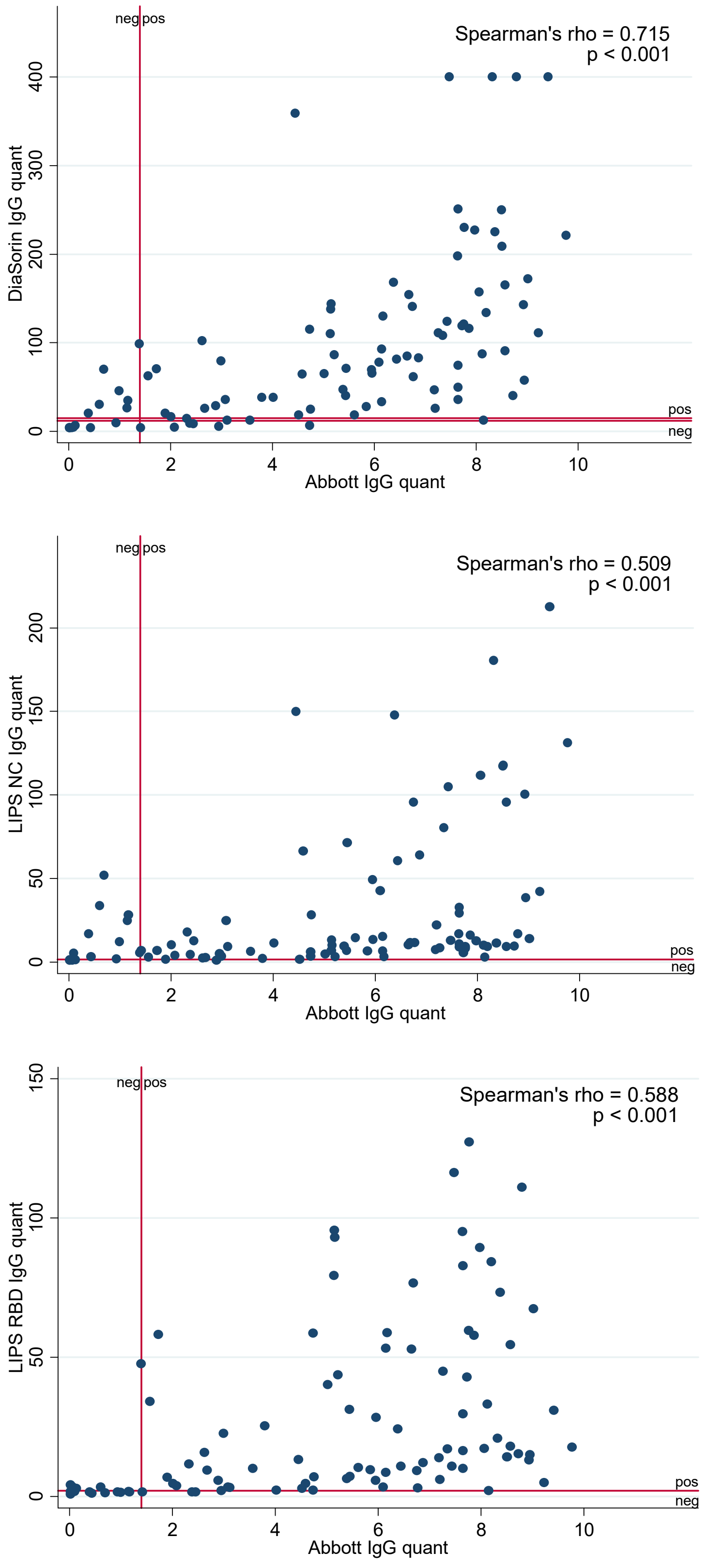

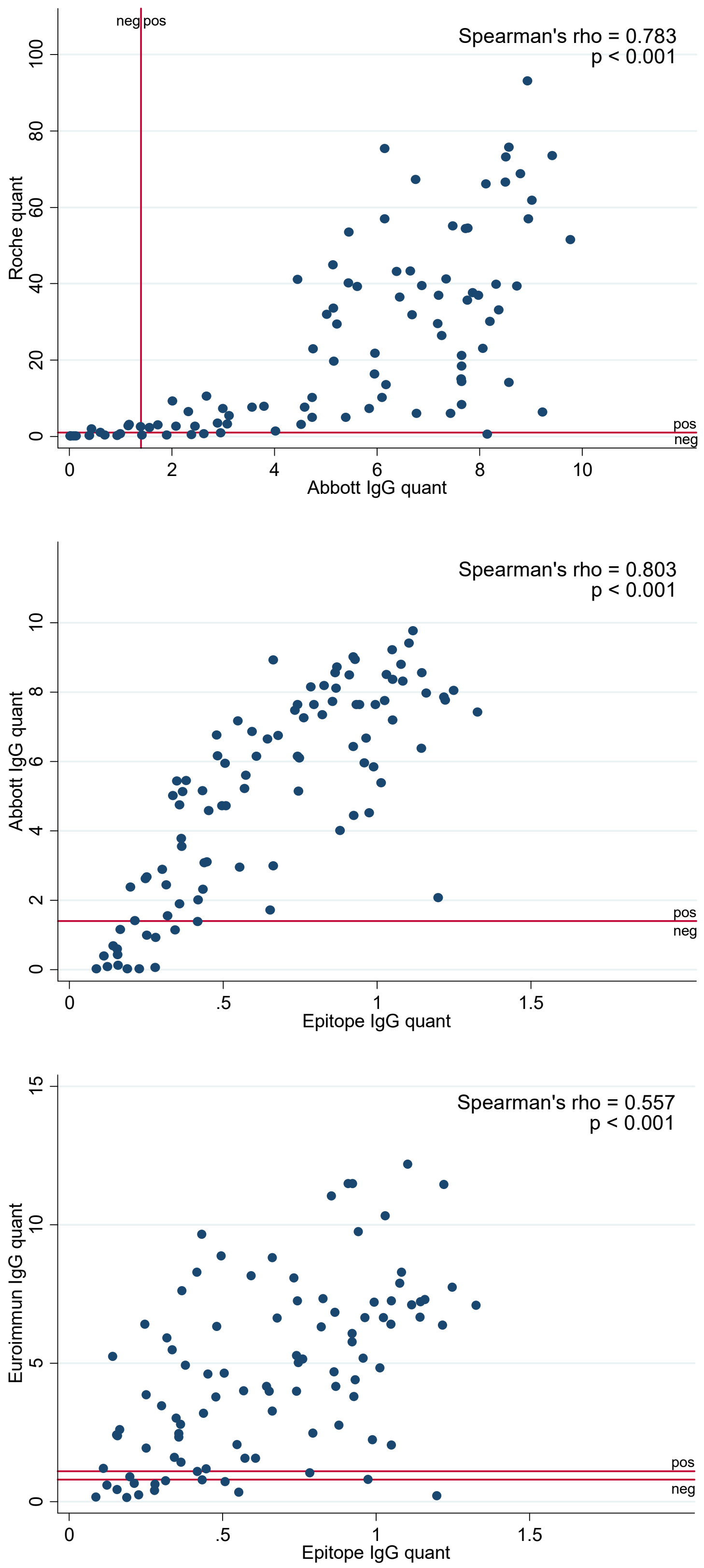

neg pos
Spearman's rho = 0.783
p < 0.001
Roche quant
Abbott IgG quant
pos
neg
Spearman's rho = 0.803
p < 0.001
Abbott IgG quant
Epitope IgG quant
pos
neg
Spearman's rho = 0.557
p < 0.001
Euroimmun IgG quant
Epitope IgG quant
pos
neg

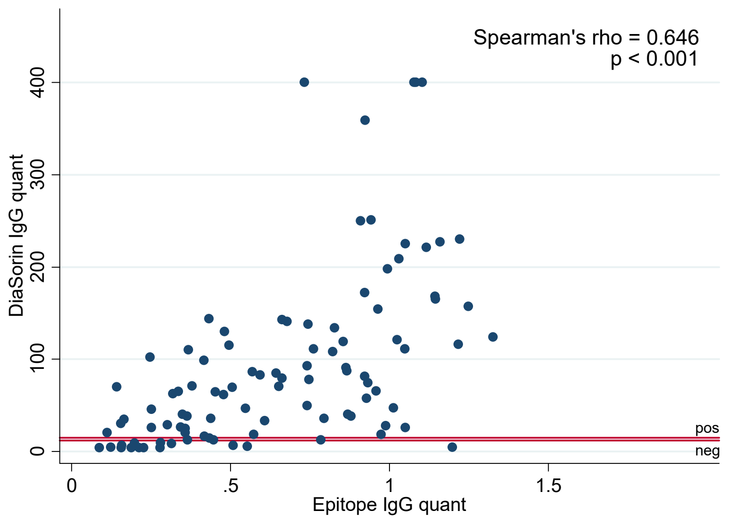

Spearman's rho = 0.646
p < 0.001
DiaSorin IgG quant
Epitope IgG quant
pos
neg

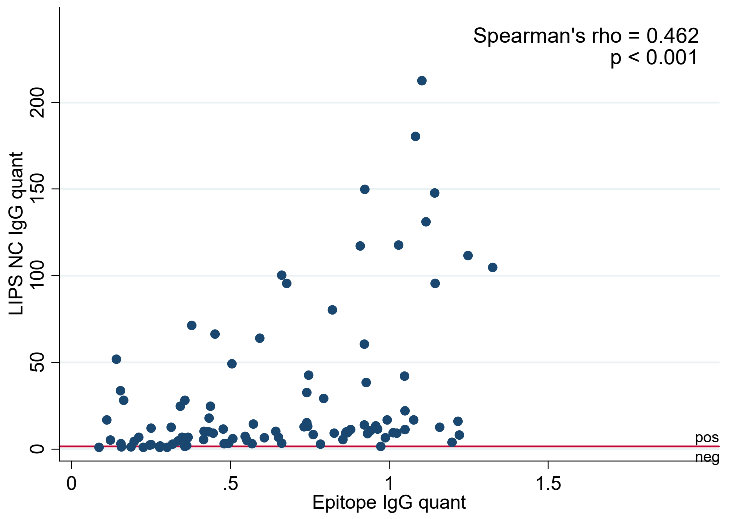

Spearman's rho = 0.462
p < 0.001
LIPS NC IgG quant
Epitope IgG quant
pos
neg

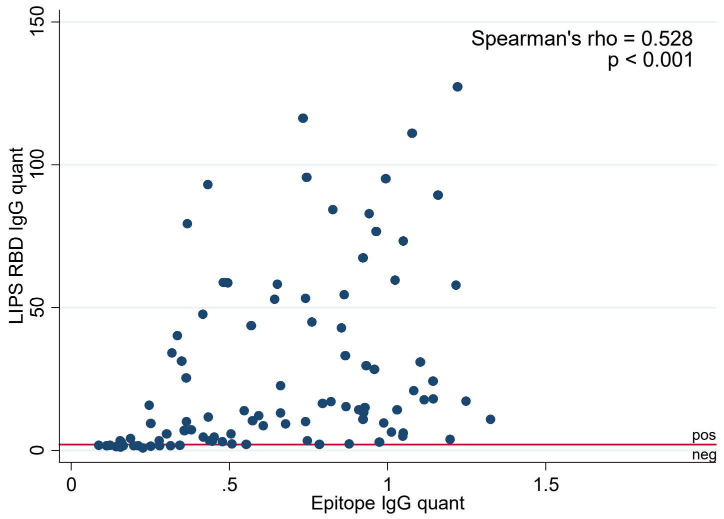

Spearman's rho = 0.528
p < 0.001
LIPS RBD IgG quant
Epitope IgG quant
pos
neg

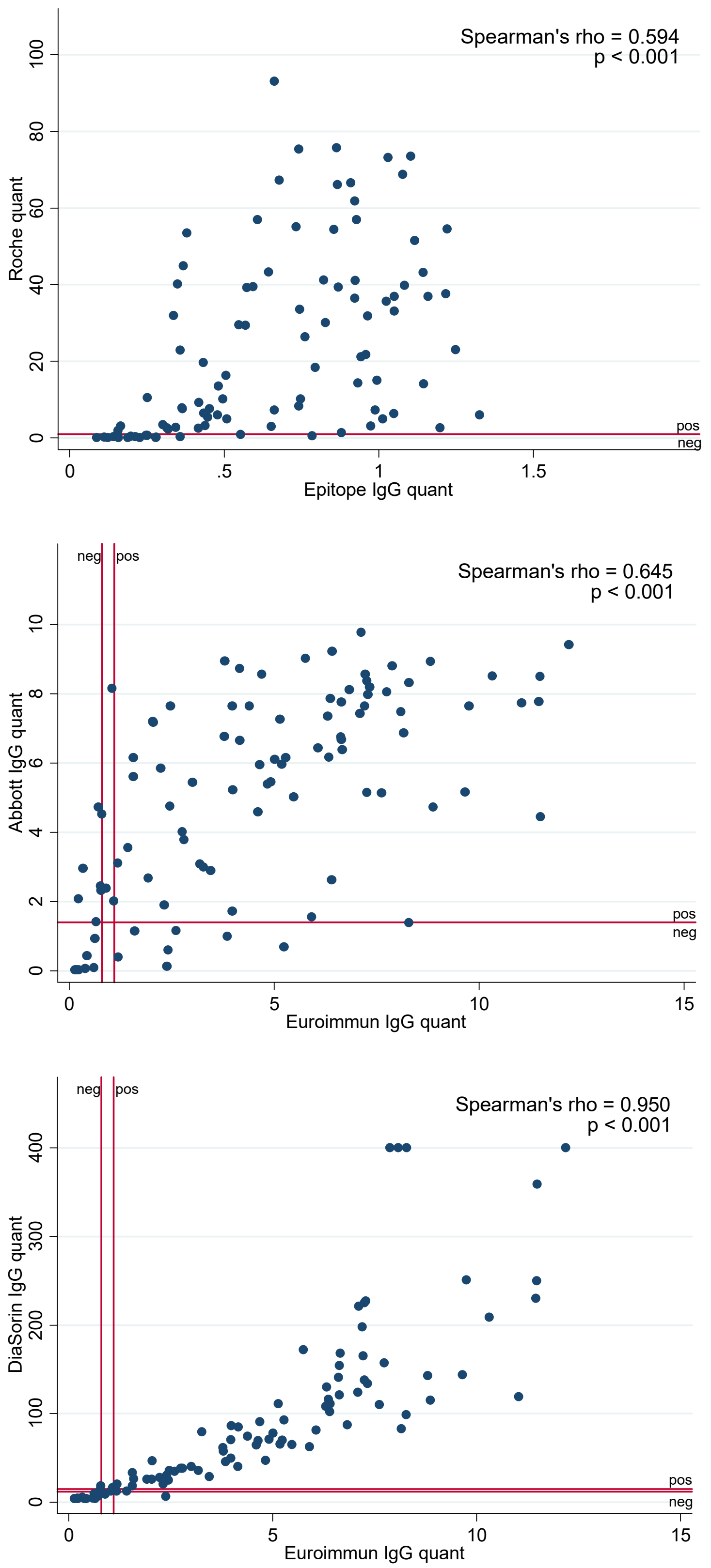
Spearman's rho = 0.594
p < 0.001
Roche quant
Epitope IgG quant
pos
neg
0
20
40
60
80
100
0
.5
1
1.5
Spearman's rho = 0.645
p < 0.001
Abbott IgG quant
Euroimmun IgG quant
neg
pos
0
2
4
6
8
10
0
5
10
15
Spearman's rho = 0.950
p < 0.001
DiaSorin IgG quant
Euroimmun IgG quant
0
100
200
300
400

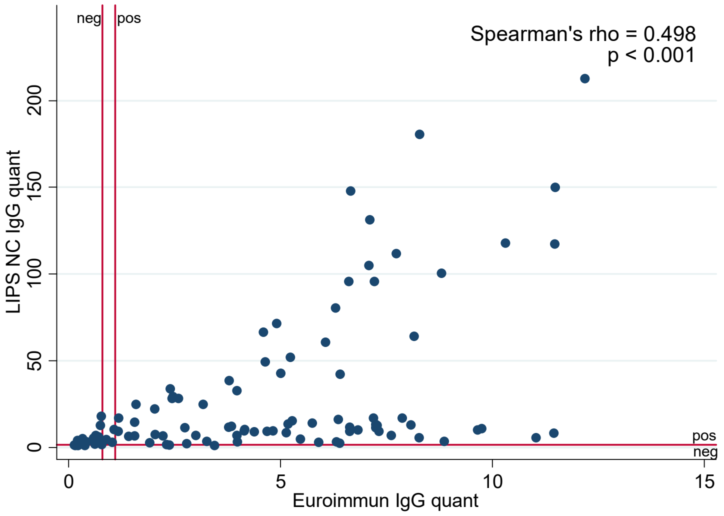
neg
pos
Spearman's rho = 0.498
p < 0.001
LIPS NC IgG quant
0
50
100
150
200
0
5
10
15
Euroimmun IgG quant
pos
neg

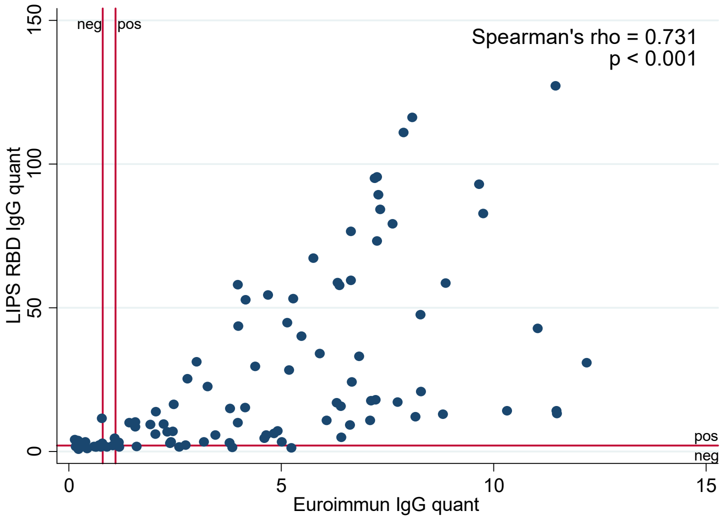
neg
pos
Spearman's rho = 0.731
p < 0.001
LIPS RBD IgG quant
0
50
100
150
0
5
10
15
Euroimmun IgG quant
pos
neg

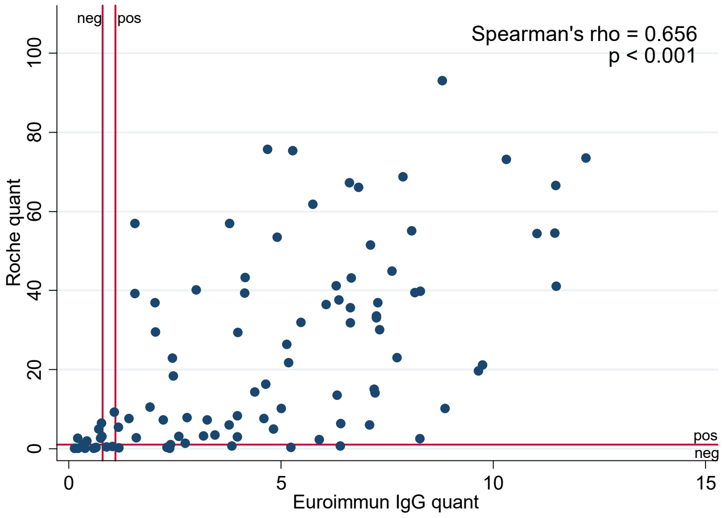
neg
pos
Spearman's rho = 0.656
p < 0.001
Roche quant
0
20
40
60
80
100
0
5
10
15
Euroimmun IgG quant
pos
neg

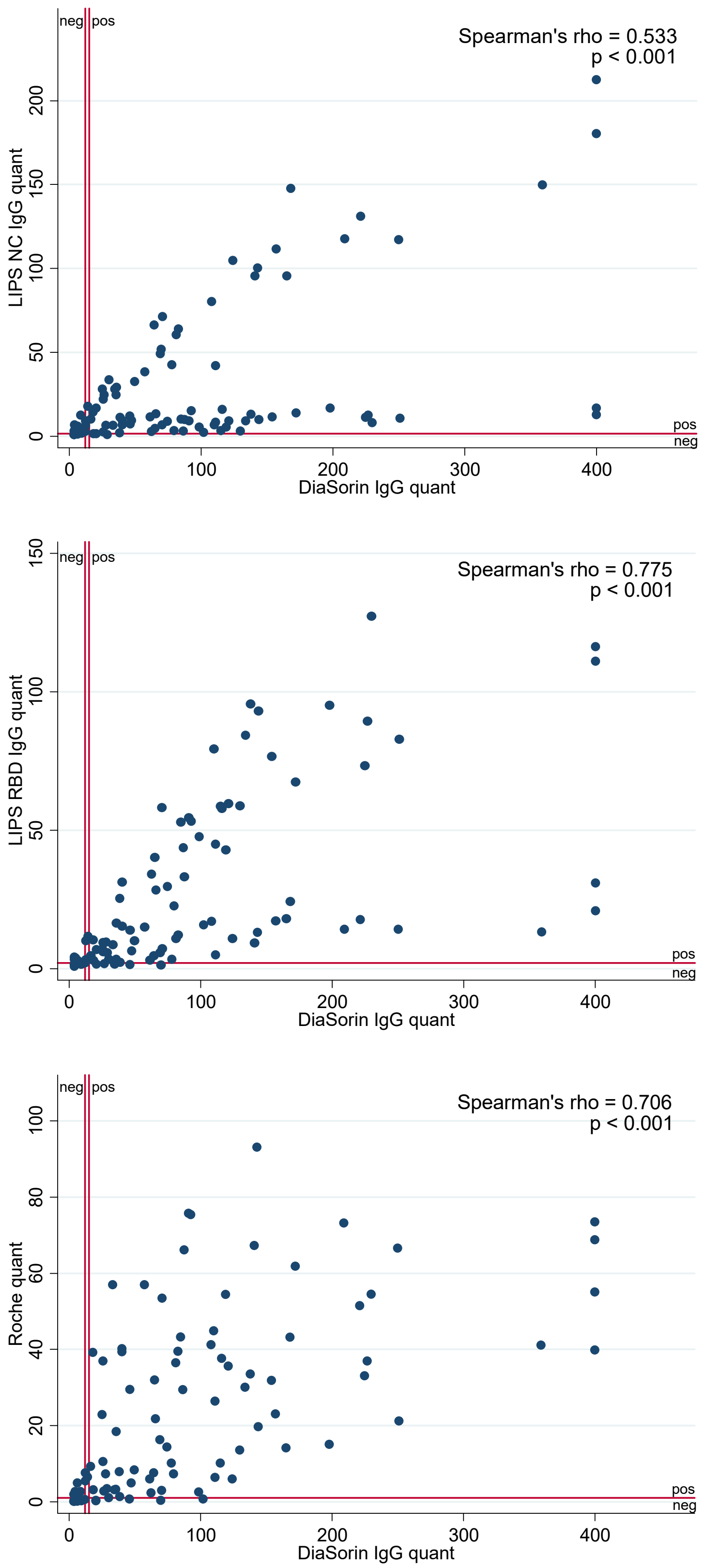
neg
pos
Spearman's rho = 0.533
p < 0.001
LIPS NC IgG quant
DiaSorin IgG quant
neg
pos
Spearman's rho = 0.775
p < 0.001
LIPS RBD IgG quant
DiaSorin IgG quant
neg
pos
Spearman's rho = 0.706
p < 0.001
Roche quant
DiaSorin IgG quant

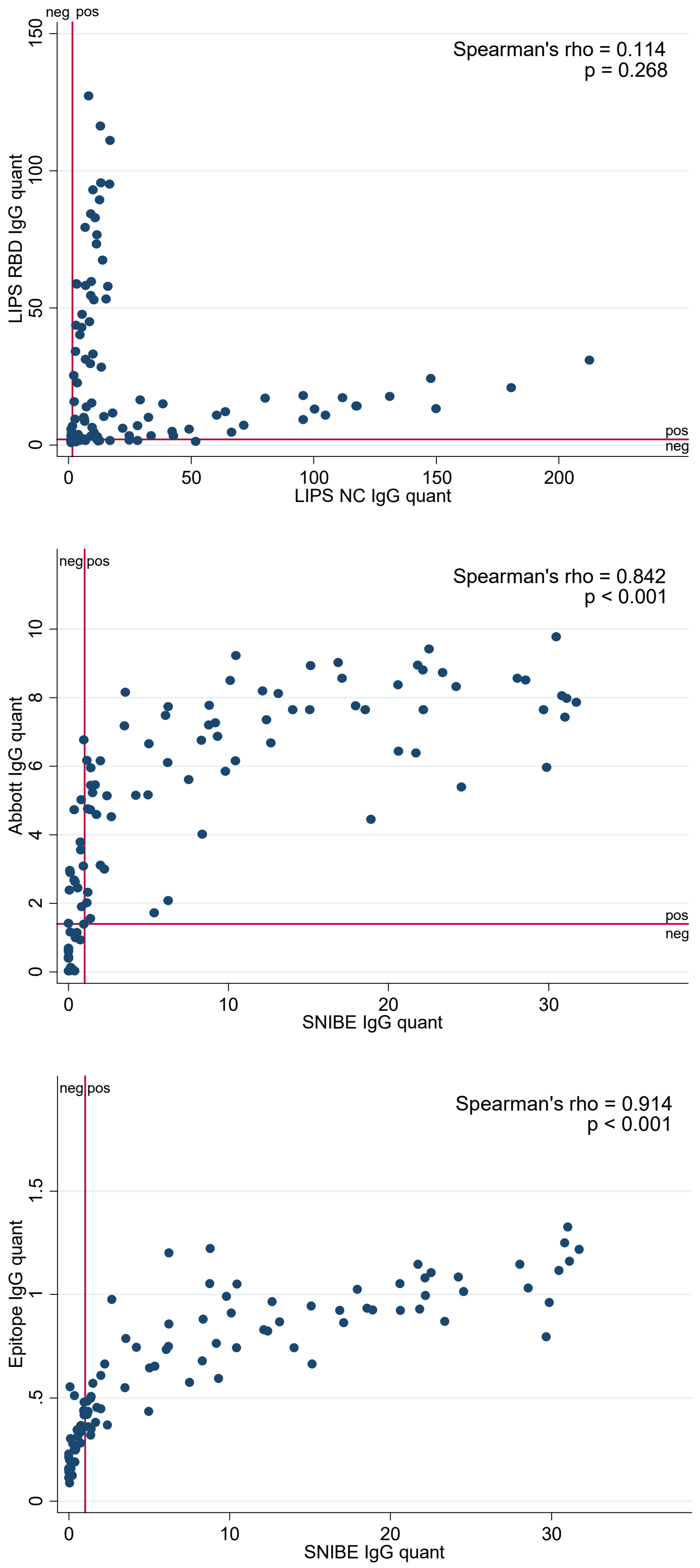

neg pos
Spearman's rho = 0.114
p = 0.268
LIPS RBD IgG quant
LIPS NC IgG quant
pos
neg
0
50
100
150
200
Spearman's rho = 0.842
p < 0.001
Abbott IgG quant
SNIBE IgG quant
0
10
20
30
Spearman's rho = 0.914
p < 0.001
Epitope IgG quant
SNIBE IgG quant

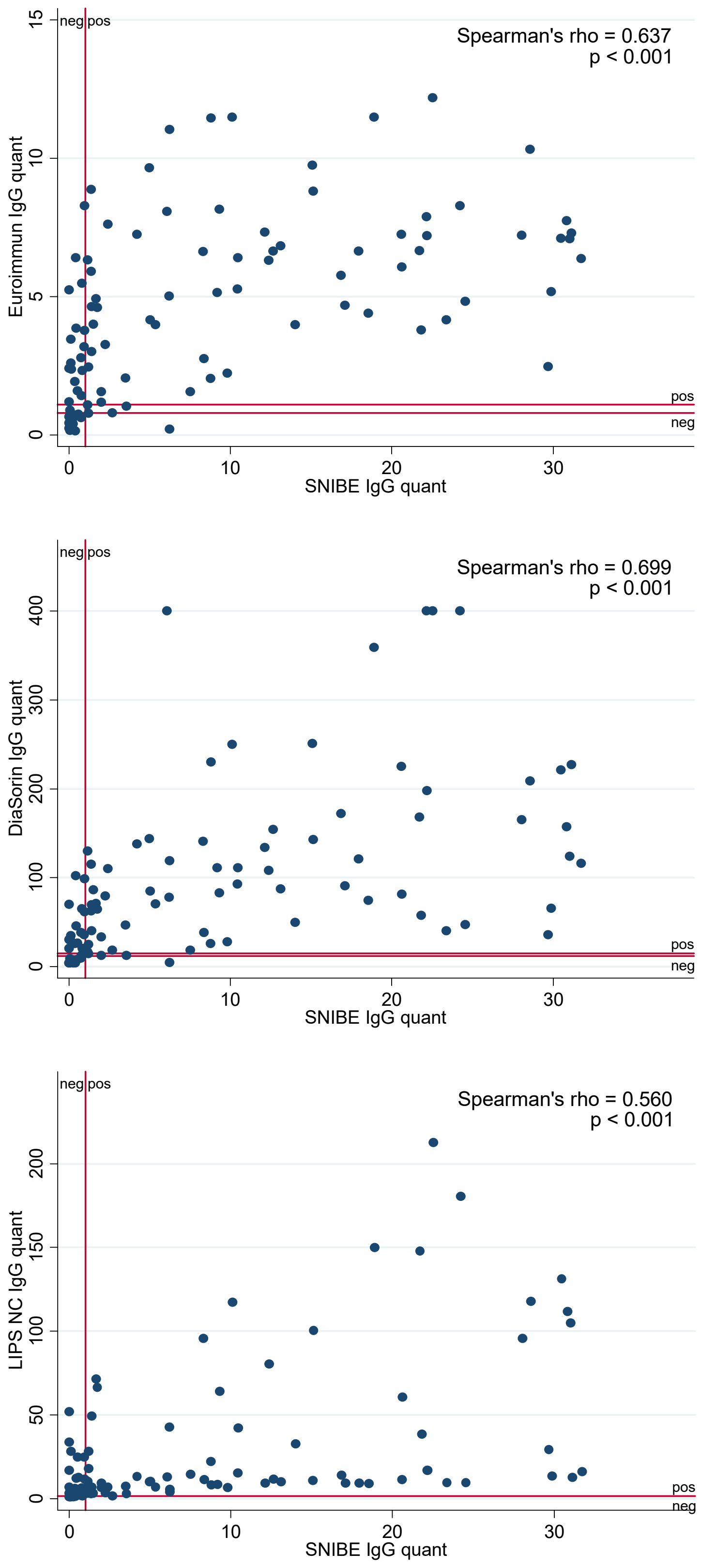

neg
pos
Spearman's rho = 0.637
p < 0.001
Euroimmun IgG quant
0
5
10
15
0
10
20
30
SNIBE IgG quant
neg
pos
Spearman's rho = 0.699
p < 0.001
DiaSorin IgG quant
0
100
200
300
400
SNIBE IgG quant
neg
pos
Spearman's rho = 0.560
p < 0.001
LIPS NC IgG quant
0
50
100
150
200
SNIBE IgG quant

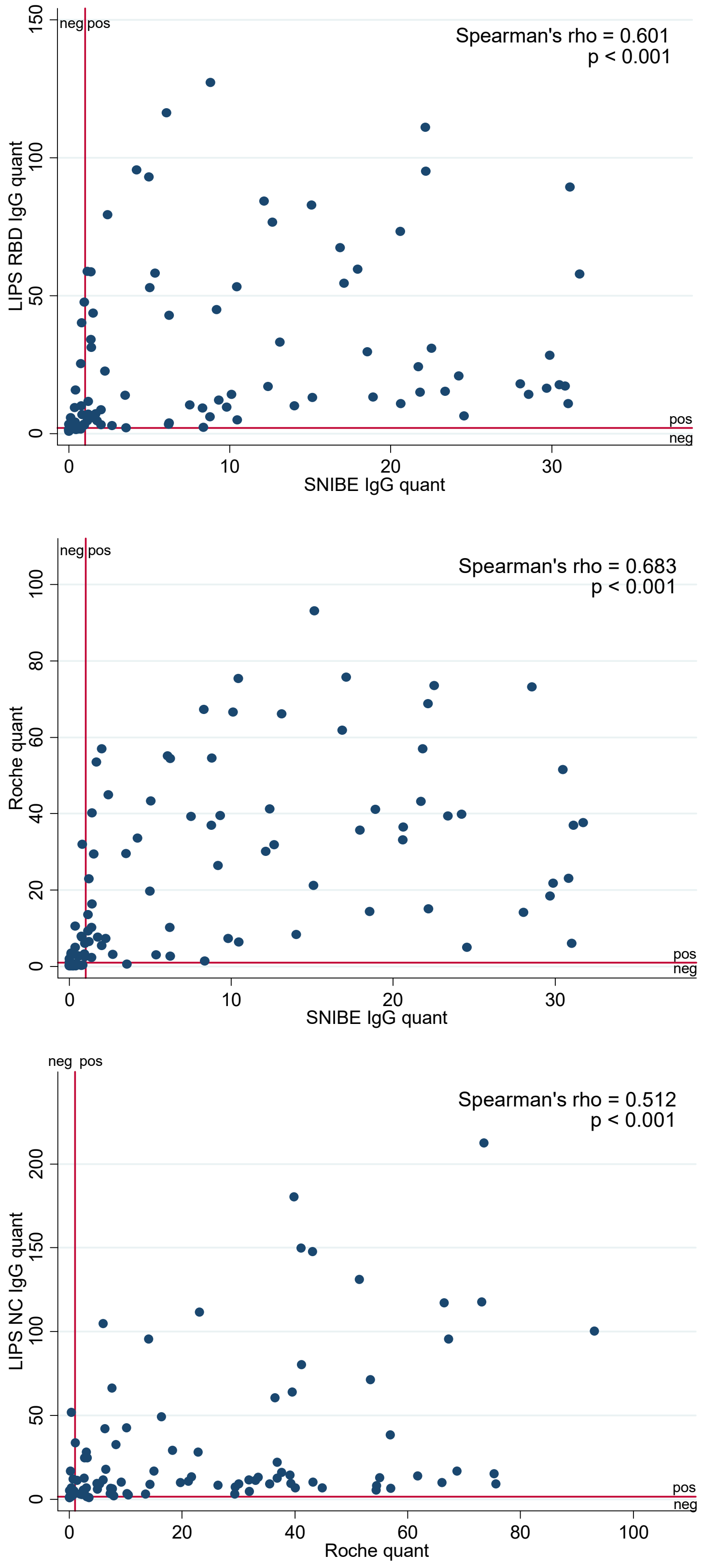
neg
pos
Spearman's rho = 0.601
p < 0.001
LIPS RBD IgG quant
0
50
100
150
pos
neg
0
10
20
30
SNIBE IgG quant
neg
pos
Spearman's rho = 0.683
p < 0.001
Roche quant
0
20
40
60
80
100
pos
neg
0
10
20
30
SNIBE IgG quant
neg
pos
Spearman's rho = 0.512
p < 0.001
LIPS NC IgG quant
0
50
100
150
200
pos
neg
0
20
40
60
80
100
Roche quant

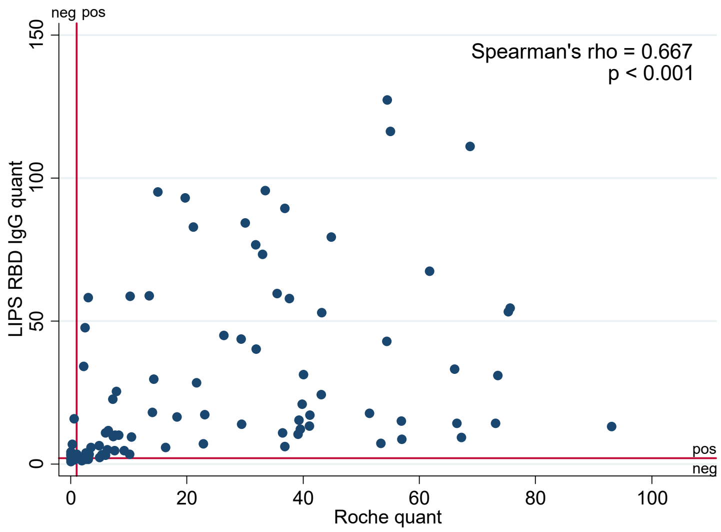
neg pos
Spearman's rho = 0.667
p < 0.001
LIPS RBD IgG quant
Roche quant
pos
neg
0
50
100
150
0
20
40
60
80
100

...
